# Prolonging the Warm-Up Effect by Using Additional Respiratory Dead Space Volume After the Cessation of Warm-Up Exercise

**DOI:** 10.3390/jcm14197049

**Published:** 2025-10-06

**Authors:** Paulina Hebisz, Rafał Hebisz, Natalia Danek

**Affiliations:** Department of Physiology and Biomechanics, Faculty of Physical Education and Sport, Wroclaw University of Health and Sport Sciences, 51-612 Wroclaw, Poland; paulina.hebisz@awf.wroc.pl (P.H.); rafal.hebisz@awf.wroc.pl (R.H.)

**Keywords:** warm-up, oxygen cost of exercise, phosphocreatine resynthesis

## Abstract

**Background**: After a warm-up and before the start of sports competition, athletes often take a break. During this break, the effects of the warm-up (e.g., capillary vasodilation) may diminish. The aim of this study was to compare cardiorespiratory responses during high-intensity physical exercise, either preceded or not preceded by post-warm-up breathing, using an additional respiratory dead space volume mask (ARDSv). **Methods**: The study included 20 trained cyclists. Each participant completed two 3 min tests at an intensity of 110% of their maximal power, determined during a progressive test. A standardised warm-up preceded each 3 min test. Following the warm-up, there was an 8 min passive rest period. During this break, participants either breathed using ARDSv or breathed normally (non-ARDSv). The volume of the ARDSv mask was 1000 mL. Cardiorespiratory parameters were measured during the tests, including mean: oxygen uptake (VO_2_av), respiratory exchange ratio (RERav), respiratory rate (RRav), tidal volume (TVav), stroke volume (SVav), and rating of perceived exertion (RPE). **Results**: VO_2_peak was higher in participants breathing using ARDSv compared to non-ARDSv (4.22 ± 0.40 [CI: 4.03–4.41] vs. 3.98 ± 0.42 [CI: 3.79–4.18]; *p* = 0.002; t = 3.56; d = 0.585). Additionally, RERav (1.08 ± 0.06 [CI: 1.06–1.11] vs. 1.13 ± 0.06 [CI: 1.11–1.16]; *p* = 0.008; t = 2.96; d = 0.833) and RPE (18.0 ± 1.7 [CI: 17.3–18.8] vs. 18.9 ± 1.1 [CI: 18.4–19.4]; *p* = 0.009; Z = 2.61; r = 0.583) were lower in participants breathing using ARDSv compared to non-ARDSv. **Conclusions**: Breathing using ARDSv between warm-up and high-intensity exercise increases oxygen uptake and reduces perceived exertion, likely through peripheral mechanisms. These effects suggest practical applications in competitive sports and provide directions for further mechanistic research.

## 1. Introduction

In many endurance sports disciplines, athletes perform high-intensity exercise during the first several minutes of a race [1,2]. This is associated with the competition for better positioning following the start [3]. Furthermore, in sports such as cross-country skiing, biathlon, mountain biking, and cyclocross, athletes encounter uphill segments, which increase the intensity of physical effort [2,4]. Studies by Næss et al. [1] and Granier et al. [3] showed that during more than 20% of a mountain bike race, muscular power exceeds maximal aerobic power, while heart rate reaches approximately 92% of maximum heart rate. Similar heart rate values have been recorded during mass start races in cross-country skiing, biathlon, running, and orienteering [5,6,7], with running speeds repeatedly exceeding those attainable at VO_2_max [8]. During very intense exercise, an oxygen deficit occurs [9], accompanied by increased anaerobic glycolytic metabolism and production of hydrogen ions and phosphates [10]. These factors impair physical work capacity [11]. Blood lactate concentration, which exceeds 7 mmol·L^−1^ during simulated cross-country skiing or mountain bike races [8,12], serves as an indirect marker of glycolytic metabolism engagement. To limit oxygen deficit during high-intensity exercise, warm-up exercises are performed [13]. Warm-up increases blood flow to muscle capillaries, muscle temperature, oxidative enzyme activity, motor unit recruitment, and lactic acidosis level, enabling enhanced aerobic metabolism during subsequent high-intensity exercise [14,15].

Before a mass start race, athletes undergo a starting line formation. Our measurements indicate that during Mountain Bike World Cup competitions, this procedure lasts from 6 to 9 min. During this time, athletes’ physical activity is limited. McGowan et al. [15] pointed out that low physical activity immediately before a race may contribute to the decline of warm-up effects. Cessation of activity causes vasoconstriction in muscle blood vessels, reducing blood flow. Thus, during the starting line procedure, warm-up effects may fade. Various countermeasures have been investigated to mitigate the decline in warm-up effects, but scientific findings are inconsistent. For example, the use of post-activation potentiation (PAP) immediately before a 1-km cycling time trial did not improve performance [16], whereas its use before a 4-km time trial significantly reduced completion time [17]. Similarly, the use of external heating devices before a 30 s sprint did not improve performance [18]. Such inconsistency in results highlights the need to find alternative strategies that can delay the decline in physiological adaptations induced by warm-up. Sperlich et al. [19] suggested that repeated apnoea before a 4 km time trial, causing temporary hypoxia with CO_2_ accumulation, would stimulate the spleen to contract in order to increase haemoglobin concentration and blood oxygen capacity. Another method is the use of additional dead space respiratory volume, which provides a practical means of elevating arterial CO_2_ partial pressure during breathing. Carbon dioxide (CO_2_) is one of the factors involved in the mechanisms of capillary vasodilation in muscles [20]. CO_2_ exerts a vasodilatory effect [21] primarily through pH-mediated activation of potassium channels and subsequent relaxation of vascular smooth muscle [22]. The resultant vasodilation, triggered by elevated partial pressures of CO_2_, reduces peripheral vascular resistance and augments both stroke volume and cardiac output [23,24,25]. The use of additional respiratory dead space volume (ARDSv) when breathing can easily elevate the partial pressure of CO_2_ in the blood. Studies by Smolka et al. [26], Danek et al. [27], and Zaton et al. [28] showed that increasing respiratory dead space volume by 1000–1200 mL, using a breathing mask connected to a tube, raises exercise-induced arterialised blood pCO_2_ by 3–7 mmHg. Therefore, it is possible that breathing using ARDSv during the interval between warm-up and high-intensity exercise enhances capillary blood flow in muscles, consequently allowing for more oxygen-demanding exercise (i.e., higher oxygen uptake). Previous research [26,27] also observed a decrease in blood pH following exercise involving breathing using ARDSv. The extent of homeostasis disruption is associated with commonly used measures of perceived exertion or well-being [29,30]. Therefore, if the reduced pH effect persists following breathing using ARDSv, it may increase the risk of deteriorated well-being during intense physical exertion.

The aim of this study was to compare cardiorespiratory responses during high-intensity physical exercise with and without post-warm-up breathing using ARDSv. An additional aim was to compare perceived exertion following high-intensity exercise under both conditions. We hypothesised that preceding high-intensity exercise with breathing using an additional 1000 mL of dead space volume would allow for exercise at a higher oxygen uptake. We also hypothesised that this approach would result in greater perceived exertion compared to exercise not preceded by breathing restriction.

## 2. Materials and Methods

### 2.1. Participants

The study involved 20 trained male cyclists aged 18–22 years, whose characteristics are presented in Table 1, whose fitness level was classified as level 3 on the five-point scale developed by De Pauw et al. [31]. The sample size was determined based on the assumption of achieving moderate statistical effects using the paired Student’s *t*-test. Using G-Power software, it was calculated that the minimum required sample size was 19 participants. Each participant had at least 3 years of cycling training experience, defined as engaging in aerobic endurance training for a minimum of 450 h annually, participating in at least 10 cycling races per year, and completing progressive exercise testing at least twice a year (including once within the two months before the study).

Each participant was informed about the study methods and provided written informed consent to participate. The study was conducted following the Declaration of Helsinki, and approval was obtained from the Institutional Ethics Committee for Scientific Research (protocol code: 14/2024; date of approval: 29 May 2024).

### 2.2. Experimental Procedure

**Figure 1:**
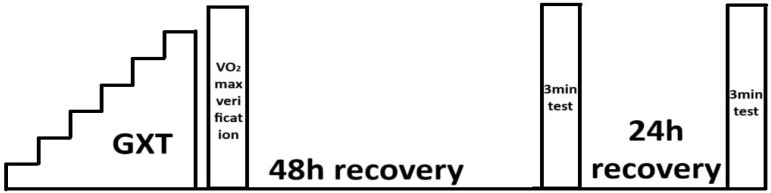
Diagram illustrating the study protocol.

Participants refrained from intense physical exertion for 48 h before the first exercise test. During this period, they were not allowed to perform any activity lasting more than 60 min or exceeding the intensity of the first ventilatory threshold (as determined in the progressive test performed within two months before the experiment). During the first visit to the exercise laboratory, baseline assessments were conducted, including anthropometric measurements (body mass, body height). Additionally, blood morphology parameters were analysed using the ABX Micros OT 16 analyser (Horiba Group, Kyoto, Japan), and blood pressure was measured using an Omron device (Omron Healthcare, Hoofddorp, The Netherlands) to assess the ability to perform the study protocol exercises. Individuals with hypertension, excessive blood viscosity, or leucocytosis were excluded from the study. None of the recruited participants exhibited these conditions. All individuals included in the experiment had received clearance for cycling training from a sports medicine physician. Following baseline testing, participants performed a progressive test with a verification phase. After completing the progressive test, cyclists rested for 48 h. Then, during the next laboratory visit, they performed a 3 min exercise test. Another 3 min test was performed after 24 h of rest. A 15 min warm-up preceded each 3 min exercise test. The tests differed in the breathing method during the 8 min passive break between warm-up and exercise. Before the 3 min test, participants either breathed using ARDSv or breathed normally. A crossover design protocol was used to determine the test order. All tests were conducted on a Lode Excalibur Sport cycle ergometer (Lode BV, Groningen, The Netherlands). The study design is illustrated in Figure 1.

#### 2.2.1. Progressive Test with Verification Phase

The experimental procedure included an incremental (step) test starting at 40 W, with the load increasing by 40 W every 3 min until the participant refused to continue or cadence dropped below 60 rpm. The maximal power output (Pmax) was defined as the final completed workload, adjusted by subtracting 0.22 W for every second short of completing the full stage, in line with the methodology used by Hebisz and Hebisz [32]. After completing the progressive test, cyclists performed a 10 min cool-down at 20 W, followed by the verification phase, during which they exercised at 110% of Pmax, as described in previous studies [33]. As with the progressive test, the verification phase continued until the participant refused or cadence fell below 60 rpm. Respiratory parameters were continuously measured using the Quark ergospirometer (Cosmed, Rome, Italy), including: minute ventilation (VE), oxygen uptake (VO_2_), carbon dioxide output (VCO_2_), respiratory exchange ratio (RER), respiratory rate (RR), tidal volume (TV), end-tidal partial pressure of oxygen (PETO_2_), and end-tidal partial pressure of carbon dioxide (PETCO_2_). The respiratory data were averaged in 30 s intervals to determine maximal oxygen uptake (VO_2_max). The first ventilatory threshold (VT1) was identified at the point of nonlinear increases in VE/VO_2_ and PETO_2_, and the second ventilatory threshold (VT2) at the point of nonlinear increases in VE/VCO_2_ and a decrease in PETCO_2_ [34]. Thresholds were independently determined by two assessors. In case of discrepancy, a third assessor made the final determination.

#### 2.2.2. The 3 Min Non-ARDSv Test

A 15 min warm-up preceded this test. During the warm-up, participants exercised at VT1 intensity for 5 min, followed by 10 min at a workload calculated using the formula P = (VT1 + VT2)/2. After the warm-up, the athletes rested for 8 min. The duration of rest was based on observations from Mountain Bike World Cup races. Following the rest period, participants performed a 3 min high-intensity exercise bout at 110% of Pmax. The intensity was determined based on data from Granier et al. [3], which described effort intensities during the initial minutes of mountain bike races, as well as the authors’ analyses of race data from the Mountain Bike World Cup.

As in the progressive test, respiratory and cardiovascular parameters were measured using the Quark ergospirometer. Mean values were calculated for the entire 3 min test (VEav, VO_2_av, VCO_2_av, RERav, RRav, TVav), as well as peak values (VEpeak, VO_2_peak, VCO_2_peak, RERpeak, RRpeak, TVpeak), based on data averaged in 30 s intervals. Oxygen uptake during the 1st, 2nd, 3rd, 4th, and 5th minutes of recovery (VO_2_rec-1min, VO_2_rec-2min, VO_2_rec-3min, VO_2_rec-4min, VO_2_rec-5min) was calculated, along with the mean RER during the 5 min recovery period (RERrec-av) and the peak RER value for recovery (RERrec-tot). Stroke volume (SV) and heart rate (HR) were measured using the PhysioFlow Enduro impedance cardiograph (Manatec Biomedical, France). Subsequently, mean values for stroke volume (SVav), heart rate (HRav), and cardiac output (COav) were calculated, as well as peak values (SVpeak, HRpeak, COpeak), again based on 30 s averaged data. Immediately after the test, systolic (SBP) and diastolic (DBP) blood pressures were measured using an Omron upper-arm blood pressure monitor (Omron Healthcare, Hoofddorp, The Netherlands). Stroke volume based on blood pressure (SVBP) was calculated using the Starr formula [35]. Following the 3 min test, each participant rated their perceived exertion (RPE) using the Borg scale, by indicating a value from 6 (very light effort) to 20 (maximal exertion) [30,36].

#### 2.2.3. The 3 Min ARDSv Test

**Figure 2:**
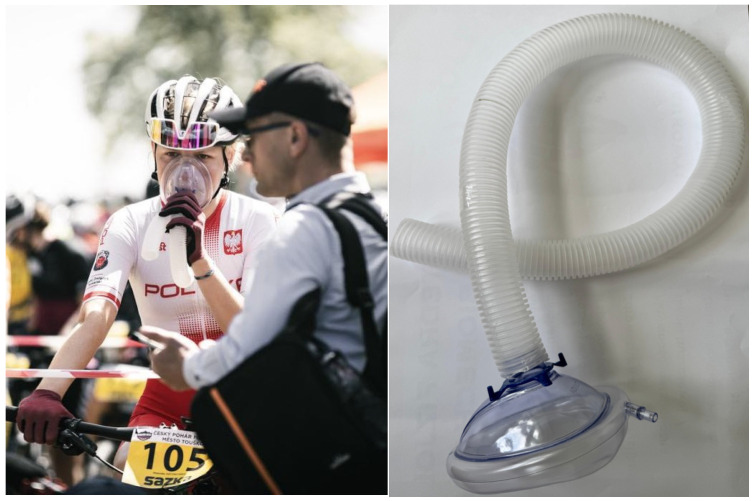
Customised device increasing respiratory dead space volume (ARDSv) by 1000 mL, consisting of an Ambu mask (disposable Ambu^®^ face masks) connected by a ribbed tube with a diameter of 2.8 cm.

The test was conducted according to the same protocol as the 3 min non-ARDSv test. The only difference was that during the 8 min passive break between the warm-up and the exercise test at 110% Pmax, the cyclists breathed using an additional 1000 mL of respiratory dead space. The ARDSv device was constructed using an Ambu mask (Ambu^®^ Disposable Face Masks) connected to a 2.8 cm diameter ribbed tube to increase the anatomical dead space by 1000 mL (Figure 2). The ARDSv was identical for each participant and was measured by filling the tube with water and then transferring the volume to a measuring cylinder [37]. The ARDSv device was applied to the participants’ faces immediately after completing the warm-up and removed 50 s before the onset of exercise at 110% Pmax (to allow time for fitting the ergospirometry mask and enabling participants to focus before the intense effort). Both series of tests were conducted under the same controlled conditions (temperature: 21 °C, relative humidity: 50%) in an air-conditioned exercise laboratory (PN-EN ISO 9001:2009 certified). All trials were performed at the same time of day (±1 h) to minimise circadian variability. The measurements were taken by a laboratory employee using a device calibrated before each test.

### 2.3. Statistical Analysis

The minimum number of participants (19) in the study was determined based on the assumption of a large effect size (Cohen’s d = 0.8) with a high test power (95%) in a paired *t*-test. The calculations were performed using G*Power 3.1.9.7 (Kiel, Germany). For all data, the arithmetic mean, standard deviation, and the lower and upper confidence intervals (CI) for the mean were calculated using Statistica 13.3 (StatSoft Inc., Tulsa, OK, USA) software. The Shapiro–Wilk test was employed to assess the normality of data distribution. For variables that followed a distribution similar to normal, a paired *t*-test was used to compare results between the ARDSv and non-ARDSv tests. Cohen’s d was used to determine the effect size for the paired *t*-test. Based on Cohen’s guidelines [38], we assumed that 0.2 represents a small effect size, 0.5 a medium effect size, and 0.8 a large effect size. For variables that significantly deviated from a normal distribution, the non-parametric Wilcoxon test was used. In these cases, the effect size r was calculated following the guidelines of Fritz et al. [39]. Thresholds for interpreting the effect size were defined as 0.1 for a small effect, 0.3 for a medium effect, and 0.5 for a large effect. A *p*-value of <0.05 was considered statistically significant. The Benjamini–Hochberg procedure [40] was applied to calculate the threshold value (q), below which a *p*-value was considered statistically significant. Calculations were performed separately for the exercise respiratory parameters (Table 2), recovery parameters (Table 3), and cardiovascular parameters (Table 4), in order to limit the risk of false-positive statistically significant results.

## 3. Results

The Shapiro–Wilk test showed that most respiratory parameters exhibited a distribution consistent with normality. Only RRpeak (W = 0.901; *p* = 0.045) and RERpeak (W = 0.831; *p* = 0.003) measured in the ARDSv test significantly differed from the normal distribution.

Statistically significant differences were found between the non-ARDSv and ARDSv tests in the following respiratory parameters: VO_2_peak (t = 3.56; *p* = 0.002; Cohen’s d = 0.585; medium effect), RERav (t = 2.96; *p* = 0.008; Cohen’s d = 0.833; large effect).

**Figure 3:**
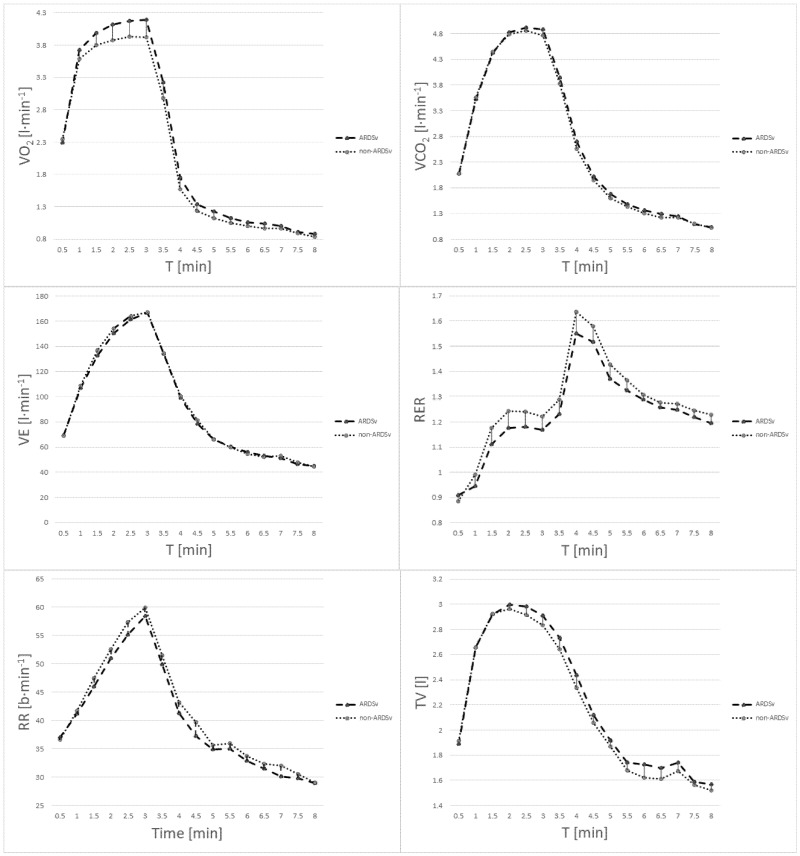
Respiratory parameters during the exercise test and recovery following the ARDSv and non-ARDSv protocols. Time 0.5–3 min represents the exercise test, 3.5–8 min corresponds to the recovery period after the exercise test.

No deviations from normal distribution were observed for respiratory recovery parameters. Statistically significant differences between the non-ARDSv and ARDSv test were found in the following: VO_2_rec-1min (t = 3.80; *p* = 0.001; Cohen’s d = 0.907; large effect), VO_2_rec-2min (t = 4.19; *p* = 0.000; Cohen’s d = 0.594; medium effect), VO_2_rec-3min (t = 3.61; *p* = 0.002; Cohen’s d = 0.444; small effect), VO_2_rec-4min (t = 2.88; *p* = 0.010; Cohen’s d = 0.374; small effect), RERrec-av (t = 2.70; *p* = 0.014; Cohen’s d = 0.500; medium effect), RERrec-peak (t = 2.68; *p* = 0.015; Cohen’s d = 0.707; medium effect). The flow of VO_2_, VCO_2_, VE, TV, RR, RER during restitution is shown in Figure 3.

RPE measured in the non-ARDSv (W = 0.831; *p* = 0.003) and ARDSv (W = 0.904; *p* = 0.049) test protocols significantly deviated from the normal distribution. Using the Wilcoxon test, statistically significant differences were found between RPE in the non-ARDSv and ARDSv tests (Z = 2.61; *p* = 0.009; r = 0.583; large effect). In this case, only 14 valid pairs were observed, as the Wilcoxon test excludes pairs with identical values.

Among the cardiovascular parameters, HRpeak (W = 0.743; *p* = 0.000), HRav (W = 0.839; *p* = 0.004), and SBP (W = 0.847; *p* = 0.004) measured during normal breathing differed significantly from the normal distribution. Similarly, HRpeak (W = 0.766; *p* = 0.000), HRav (W = 0.881; *p* = 0.018), and DBP (W = 0.832; *p* = 0.003) measured during the test preceded by breathing using the ARDSv device also deviated significantly from the normal distribution. A significant deviation from the normal distribution was also observed for RPE, both in the non-ARDSv test (W = 0.831; *p* = 0.003) and in the test preceded by breathing using ARDSv (W = 0.904; *p* = 0.049). No statistically significant differences were observed between the non-ARDSv and ARDSv exercise protocols for any of the cardiovascular parameters.

## 4. Discussion

The most important result of the presented study is the indication of the differences in oxygen uptake and respiratory exchange ratio between two 3 min exercise tests in participants who applied ARDSv and breathed normally after the warm-up. In maximal exercise lasting over one minute, aerobic metabolism predominates [41]. However, anaerobic metabolism plays a significant role during high-intensity efforts lasting several minutes [42]. An indirect indicator of glycolytic anaerobic metabolism is RER exceeding 1, as VCO_2_ surpassing VO_2_ is generated in the process of hydrogen ion buffering [43]. In the 3 min effort performed following breathing using ARDSv, significantly lower RER values and higher VO_2_ values were observed compared to the effort after normal breathing. These findings suggest that the anaerobic energy cost during exercise preceded by breathing using ARDSv was lower compared to normal breathing. Simultaneously, the aerobic energy cost was higher in the participants who breathed using ARDSv compared to those who breathed normally. This effect may be advantageous in the context of sports competition, as intense anaerobic metabolism during exertion leads to acid-base imbalance [44], rapid glycogen depletion [45], and production of inorganic phosphates [42]. These consequences of anaerobic metabolism are associated with reduced work capacity [42]. Further research is necessary to assess the impact of breathing using ARDSv on exercise capacity. The test procedure used in this study involved performing exercise at a fixed intensity and duration. Based on the test conducted here, it is not possible to definitively determine or generalise changes in exercise capacity. To evaluate exercise capacity, future studies should incorporate time-to-exhaustion protocols at constant power or fixed-duration tests with voluntarily selected intensity.

The results show that during the first four minutes of recovery, oxygen uptake was higher in the participants breathing using ARDSv compared to those breathing normally. The greatest difference was observed during the first minute of recovery. Excess post-exercise oxygen uptake within 3–5 min post-exercise is associated with phosphocreatine resynthesis in mitochondria via oxidative phosphorylation [46,47,48]. Data presented by Zoladz et al. [49] indicate that nearly full recovery of muscle phosphocreatine occurs within four minutes of recovery. Moreover, approximately 70% of phosphocreatine is replenished within 30 s of exercise cessation [50]. Accordingly, the differences observed in this study may reflect more intensive phosphocreatine resynthesis in the ARDSv test compared to the non-ARDSv test.

No differences in oxygen uptake were observed in the fifth minute of recovery between the ARDSv and non-ARDSv protocols. It is possible that phosphocreatine resynthesis was nearing completion by this time among the participants. According to Børsheim and Bahr [51], excess oxygen consumption following the completion of phosphocreatine restoration reflects glycogen synthesis using lactate as a substrate. The fact that differences in recovery oxygen uptake between the ARDSv and non-ARDSv protocols decreased with each passing minute, and disappeared entirely by the fifth minute, may indicate that the increased dead space volume did not influence glycogen resynthesis.

In response to a significant disturbance of homeostasis, the rate of perceived exertion increases [29,30]. A widely used measure of perceived exertion during and after exercise is the RPE scale [30]. In this study, RPE was lower in participants breathing using ARDSv compared to those breathing normally. This suggests that the higher intensity of aerobic metabolism in participants breathing using ARDSv may have enabled the performance of intense exercise with less disruption to acid-base balance. The results support the potential use of ARDSv to prolong the effects of the warm-up. Further studies are needed to better explore the role of ARDSv in immediate preparation for high-intensity efforts. Future investigations should assess oxygen deficit and post-exercise oxygen excess over a ~60 min period, as proposed by Jung et al. [52]. It would also be valuable to compare the acid-base balance between athletes breathing using ARDSv and those breathing normally.

According to Fick’s principle, oxygen uptake is determined by cardiac output and the arteriovenous oxygen difference [53]. In this study, stroke volume during exercise was measured via impedance cardiography, and post-exercise stroke volume was estimated using Starr’s formula [35]. Stroke volume, as measured by various methods, did not significantly differ between participants breathing using ARDSv and those breathing normally. These results suggest that the observed differences in oxygen uptake stemmed more from peripheral mechanisms than from central ones. Further studies, such as analyses of acid-base balance and arteriovenous oxygen differences, could provide a deeper understanding of these mechanisms.

The results of the study showed that respiratory parameters (RR, TV, and VE) did not differ between the protocols. These findings contrast with earlier studies on exercise with breathing using ARDSv, which indicated higher VE (66.3 ± 8.2 L·min^−1^ v. 57.8 ± 4.4 L·min^−1^) and TV (2.7 ± 0.5 L v. 2.4 ± 0.3 L) during efforts involving ARDSv compared to normal breathing [27]. The elevated respiratory parameters may have resulted from breathing using ARDSv, which increases pCO_2_ (44.0 ± 2.4 mmHg) and reduces arterialised blood pH (7.37) [27]. Elevated pCO_2_ and decreased pH stimulate chemoreceptors in the circulatory system, resulting in increased pulmonary minute ventilation [54]. Higher respiratory parameters increase respiratory work, potentially leading to competition for blood supply between locomotor and respiratory muscles, and ultimately to redistribution of blood toward the respiratory muscles [20,55]. This effect was not observed in the present study. Comparable findings were reported in sprint swimming research [56], where an ARDSv volume of 1200 mL was administered during a 20 min transitional phase between the warm-up and a 50 m front crawl time trial. The intervention resulted in a statistically significant improvement, reducing the time to complete the distance by 0.5 s; however, it did not statistically significantly increase the respiratory muscle workload. The results suggest that pre-exercise breathing using ARDSv does not increase the respiratory workload during subsequent intense exercise.

Limitations. It can be considered that the outcome of the presented study was limited by the fact that the participants were not blinded, and the results obtained were due to the occurrence of a placebo effect. A similar fact was previously reported by Woorons et al. [57] investigating the effects of hypoventilation. This problem recurs with the use of ARDSv, as it is impossible to conduct single or double-blind studies. However, although a psychological effect cannot be excluded in the present study, it should be noted that the participants were not aware of or received any information about the possible impact of the tested method.

In addition, the generalizability of the presented findings is limited, as the sample included only cyclists with a relatively homogeneous level of aerobic efficiency. A more diverse population, including individuals with different characteristics and training backgrounds, would be necessary to confirm the broader applicability of the results. Furthermore, in this study, a protocol with a predetermined length and intensity was applied. This protocol did not allow for the evaluation of work capacity. Therefore, future research should investigate protocols of fixed intensity performed until exhaustion, or protocols with a fixed duration combined with maximal voluntary intensity as applied in other studies [58,59]. Finally, as the study was conducted under controlled laboratory conditions, caution is warranted when extrapolating the results to real-world competitive settings, where additional factors such as psychological stress, environmental conditions, or race dynamics may influence performance outcomes. For example, in cross-country skiing, biathlon, cycling, etc., many competitions are held in hypoxic environments [60] or under high ambient temperatures [61]. At this stage, it remains difficult to predict the potential impact of ARDSv use in warm-up preparation under such varied environmental conditions.

### Practical Applications

During sports competitions, when a passive break occurs between the warm-up and the start of the race, there is a risk of diminished vasodilation in the muscle microvasculature and a reduction in exercise oxygen uptake. Findings from the presented study indicate that athletes and coaches may consider incorporating an additional respiratory dead space volume (ARDSv) of 1000 mL (mask and tube) during this interval, as this procedure may help sustain the physiological effects of the warm-up, increase oxygen uptake during subsequent high-intensity efforts, and reduce the respiratory exchange ratio (RER). From a practical perspective, this approach may be particularly effective before competitions that begin with high-intensity exercise, such as cycling, cross-country skiing, or biathlon events. The use of ARDSv may promote more efficient aerobic energy production and reduce reliance on anaerobic metabolism at the onset of competition, thereby delaying fatigue. These findings should be regarded as preliminary and specific to the tested protocol (1000 mL, ~8 min break), and further studies are needed to verify their generalizability across different durations, intensities, athlete populations, and gender.

## 5. Conclusions

The results obtained in the presented study represent a significant contribution to the existing knowledge on methods supporting the effectiveness of warm-up. Previous research has focused mainly on post-activation potentiation and external muscle heating. Our approach concentrated on inducing modifications in the composition of respiratory gases through the application of an increased dead space breathing volume (ARDSv). Presented results indicate that this method may have a substantial impact on energy metabolism during high-intensity exercise. Breathing using ARDSv during the interval between warm-up and intensive exercise leads to an increase in oxygen cost and a reduction in the respiratory exchange ratio during high-intensity effort, while simultaneously decreasing the subjective perception of exertion. The mechanism responsible for the increased oxygen uptake is likely peripheral (muscular) in nature, as no changes in stroke volume were observed. Based on the presented results, we conclude that ARDSv may serve as a valuable complement to warm-up before sports competitions requiring high-intensity aerobic metabolism, when the start is preceded by several minutes without significant physical activity. In the future, further research on breathing with ARDSv prior to exercises of maximal duration or maximal intensity may allow for the development of more detailed recommendations regarding warm-up strategies in endurance sports, as well as an assessment of the potential generalizability of this method across different disciplines.

## Figures and Tables

**Table 1:** Characteristics of the study participants.

Variables	$\bar{x}$ ± SD
Age [years]	20.3 ± 1.5
BM [kg]	70.1 ± 8.5
BH [m]	1.79 ± 0.07
VO_2_max [mL·kg^−1^·min^−1^]	57.7 ± 6.7
Pmax [W]	357.8 ± 43
Pmax [W·kg^−1^]	5.13 ± 0.55

BM—body mass; BH—body height; VO_2_max—maximal oxygen uptake; Pmax—maximal power output in the progressive test; $\bar{x}$—arithmetic mean; SD—standard deviation.

**Table 2:** Comparison of respiratory parameters obtained in the 3 min non-ARDSv test and the 3 min ARDSv test.

Variables	Non-ARDSv		ARDSv		*p* Value q Value	Cohen’s d (r)
	$\bar{x}$ ± SD	Lower CI Upper CI	$\bar{x}$ ± SD	Lower CI Upper CI		
RRpeak [b·min^−1^]	60.5 ± 11.1	55.3	59.5 ± 11.2	53.2	0.067	(0.409)
		65.7		63.7	0.025	
RRav [b·min^−1^]	49.7 ± 9.1	45.4	48.0 ± 9.4	43.6	0.028	0.184
		54.0		52.4	0.021	
TVpeak [L]	3.01 ± 0.39	2.83	3.04 ± 0.38	2.86	0.266	0.078
		3.20		3.22	0.037	
TVav [L]	2.73 ± 0.36	2.56	2.73 ± 0.34	2.57	0.996	0.000
		2.89		2.88	0.050	
VEpeak [L·min^−1^]	170.3 ± 24.6	158.7	168.2 ± 21.7	158.1	0.341	0.091
		181.8		178.4	0.042	
VEav [L·min^−1^]	135.4 ± 20.0	126.0	130.9 ± 17.5	122.7	0.086	0.239
		144.8		139.1	0.029	
VO_2_peak [L·min^−1^]	3.98 ± 0.42	3.79	4.22 ± 0.40	4.03	0.002 *	0.585
		4.18		4.41	0.004	
VO_2_av [L·min^−1^]	3.60 ± 0.38	3.42	3.75 ± 0.35	3.58	0.023	0.411
		3.78		3.91	0.017	
VCO_2_peak [L·min^−1^]	4.91 ± 0.43	4.71	4.97 ± 0.44	4.76	0.168	0.138
		5.11		5.18	0.033	
VCO_2_av [L·min^−1^]	4.13 ± 0.42	3.94	4.11 ± 0.37	3.93	0.654	0.051
		4.33		4.28	0.046	
RERpeak	1.26 ± 0.07	1.23	1.19 ± 0.07	1.16	0.016	(0.539)
		1.30		1.22	0.012	
RERav	1.13 ± 0.06	1.11	1.08 ± 0.06	1.06	0.0081 *	0.833
		1.16		1.11	0.0083	

RR—respiratory rate (breaths per minute); VE—pulmonary ventilation; TV—tidal volume; VO_2_—oxygen uptake; VCO_2_—carbon dioxide output; RER—respiratory exchange ratio (ratio of CO_2_ output to O_2_ uptake); av—mean value during 3 min test at 110% maximal aerobic power; peak—peak value during 3 min test at 110% maximal aerobic power; non-ARDSv—test without additional respiratory dead space volume; ARDSv—test preceded by use of additional respiratory dead space volume; $\bar{x}$—arithmetic mean; SD—standard deviation; CI—confidence interval; *p*—statistical significance; q—the *p*-value threshold calculated using the Benjamini–Hochberg procedure; Cohen’s d—effect size for paired *t*-test; (r)—effect size for Wilcoxon test; *—Statistically significant difference between 3 min tests (*p* < 0.05).

**Table 3:** Comparison of selected respiratory parameters during recovery in the 3 min non-ARDSv test and the 3 min ARDSv test.

Variables	Non-ARDSv		ARDSv		*p* Value q Value	Cohen’s d
	$\bar{x}$ ± SD	Lower CI Upper CI	$\bar{x}$ ± SD	Lower CI Upper CI		
VO_2_rec-1min [L]	2.13 ± 0.45	1.92	2.47 ± 0.28	2.34	0.001 *	0.907
		2.34		2.60	0.012	
VO_2_rec-2min [L]	1.17 ± 0.18	1.09	1.28 ± 0.19	1.20	0.000 *	0.594
		1.26		1.37	0.006	
VO_2_rec-3min [L]	1.01 ± 0.18	0.93	1.09 ± 0.18	1.01	0.002 *	0.444
		1.10		1.18	0.019	
VO_2_rec-4min [L]	0.96 ± 0.17	0.89	1.02 ± 0.15	0.95	0.010 *	0.374
		1.04		1.09	0.031	
VO_2_rec-5min [L]	0.86 ± 0.17	0.79	0.89 ± 0.16	0.82	0.208	0.182
		0.94		0.97	0.050	
RERrec-av	1.37 ± 0.10	1.32	1.32 ± 0.10	1.27	0.014 *	0.500
		1.41		1.37	0.037	
RERrec-peak	1.67 ± 0.16	1.59	1.57 ± 0.12	1.51	0.015 *	0.707
		1.74		1.63	0.044	
RPE	18.9 ± 1.1	18.4	18.0 ± 1.7	17.3	0.009	(0.583)
		19.4		18.8	0.025	

VO_2_rec-1min; rec-2min; rec-3min; rec-4min; rec-5min—oxygen uptake during the 1st, 2nd, 3rd, 4th, and 5th minutes of recovery; RERrec-av—the mean RER during the 5 min recovery period; RERrec-peak—the peak RER value for recovery; RPE—rate of perceived exertion; non-ARDSv—test without additional respiratory dead space volume; ARDSv—test preceded by use of additional respiratory dead space volume; $\bar{x}$—arithmetic mean; SD—standard deviation; CI—confidence interval from the mean value; *p*—level of statistical significance; q—the *p*-value threshold calculated using the Benjamini–Hochberg procedure; Cohen’s d—effect size for paired *t*-test; (r)—effect size for Wilcoxon test; *—Statistically significant difference between 3 min tests (*p* < 0.05).

**Table 4:** Comparison of cardiovascular parameters and perceived exertion during the 3 min non-ARDSv test and the 3 min ARDSv test.

Variables	Non-ARDSv		ARDSv		*p* Value q Value	Cohen’s d (r)
	$\bar{x}$ ± SD	Lower CI Upper CI	$\bar{x}$ ± SD	Lower CI Upper CI		
SVpeak [mL]	147.1 ± 27.3	134.3	147.0 ± 22.5	136.5	0.974	0.004
		159.9		157.6	0.044	
SVav [mL]	128.5 ± 24.6	116.9	130.6 ± 22.0	120.3	0.314	0.090
		140.0		140.9	0.011	
HRpeak [bpm]	193.3 ± 12.9	187.3	193.9 ± 11.2	188.7	0.379	(0.197)
		199.4		199.2	0.022	
HRav [bpm]	179 ± 12.7	173.1	180.5 ± 11.7	175.0	0.528	(0.118)
		184.9		185.0	0.028	
COpeak [L]	27.5 ± 4.7	25.3	27.9 ± 3.8	26.2	0.545	0.094
		29.7		29.7	0.033	
COav [L]	23.0 ± 3.8	21.2	23.5 ± 3.5	21.9	0.153	0.137
		24.8		25.2	0.006	
SBP [mmHg]	167.5 ± 27.1	154.8	171.0 ± 22.5	160.5	0.324	(0.221)
		180.2		181.5	0.017	
DBP [mmHg]	34.5 ± 24.4	23.1	35.0 ± 23.7	23.9	1.000	(0.000)
		45.9		46.1	0.050	
SV_BP_ [mL]	134.8 ± 31.8	119.9	136.0 ± 29.5	122.2	0.836	0.039
		149.7		149.8	0.039	

SV—stroke volume; HR—heart rate; CO—cardiac output; SBP—systolic blood pressure measured immediately post-exercise; DBP—diastolic blood pressure measured immediately post-exercise; SV_BP_—stroke volume estimated based on post-exercise blood pressure; av—mean value during 3 min test at 110% maximal aerobic power; peak—peak value during 3 min test at 110% maximal aerobic power; non-ARDSv—test without additional respiratory dead space volume; ARDSv—test preceded by use of additional respiratory dead space volume; $\bar{x}$—mean; SD—standard deviation; CI—confidence interval; *p*—level of statistical significance; q—the *p*-value threshold calculated using the Benjamini–Hochberg procedure; Cohen’s d—effect size for paired *t*-test; (r)—effect size for Wilcoxon test.

## Data Availability

Data will be provided on request.

## Abbreviations

The following abbreviations are used in this manuscript:

BM	Body mass
BH	Body height
VO_2_max	Maximal oxygen uptake
Pmax	Maximal power output in the progressive test
VE	Minute ventilation
VCO_2_	Carbon dioxide output
RER	Respiratory exchange ratio
RR	Respiratory rate
TV	Tidal volume
PETO_2_	End-tidal partial pressure of oxygen
PETCO_2_	End-tidal partial pressure of carbon dioxide
VT1	First ventilatory threshold
VT2	Second ventilatory threshold
VO_2_rec-1min	Oxygen uptake during the 1st minutes of recovery
VO_2_rec-2min	Oxygen uptake during the 2nd minute of recovery
VO_2_rec-3min	Oxygen uptake during the 3rd minute of recovery
VO_2_rec-4min	Oxygen uptake during the 4th minute of recovery
VO_2_rec-5min	Oxygen uptake during the 5th minute of recovery
RERrec-av	Mean RER during the 5 min recovery period
RERrec-tot	Peak RER value for recovery
SV	Stroke volume
HR	Heart rate
CO	Cardiac output
SBP	Systolic blood pressure measured immediately post-exercise
DBP	Diastolic blood pressure measured immediately post-exercise
SV_BP_	Stroke volume estimated based on post-exercise blood pressure
RPE	Rate of perceived exertion
